# Fecal Zonulin as a Non-Invasive Marker of Intestinal Permeability: Findings from a Prospective Cohort Study

**DOI:** 10.3390/medicina61091527

**Published:** 2025-08-25

**Authors:** Naomi-Adina Ciurea, Cristina Monica Pantea, Paul Grama, Irina-Bianca Kosovski, Simona Bataga

**Affiliations:** 1 Department of Internal Medicine, George Emil Palade University of Medicine, Pharmacy, Science and Technology of Targu Mures, 540139 Targu Mures, Romania; paul.grama@umfst.ro (P.G.); simona.bataga@umfst.ro (S.B.); 2 Doctoral School of Medicine and Pharmacy, George Emil Palade University of Medicine, Pharmacy, Science and Technology of Targu Mures, 540139 Targu Mures, Romania; 3 Department of Clinical Science-Internal Medicine, George Emil Palade University of Medicine, Pharmacy, Science and Technology of Targu Mures, 540139 Targu Mures, Romania; cmonicapantea@gmail.com; 4 Department of Pathophysiology, George Emil Palade University of Medicine, Pharmacy, Science and Technology of Targu Mures, 540139 Targu Mures, Romania; bianca.kosovski@umfst.ro

**Keywords:** MASLD, zonulin, fecal biomarkers, intestinal permeability, SteatoTest, FibroMax, gut–liver axis, hepatic steatosis

## Abstract

*Background and Objectives:* Metabolic dysfunction-associated steatotic liver disease (MASLD) is now widely acknowledged as belonging to the broader category of metabolic disorders, being closely associated with obesity, insulin resistance, and chronic systemic inflammation. Recent evidence indicates that in MASLD, alterations in the gut–liver axis—particularly increased intestinal permeability may serve as a crucial mechanistic link between metabolic dysfunction and hepatic steatosis. Zonulin, a physiological modulator of intestinal tight junctions, has been suggested as an indicator of compromised barrier integrity; however, its specific role in MASLD remains to be fully elucidated. *Materials and Methods:* We conducted a prospective observational study including 52 adult patients diagnosed with MASLD. Hepatic steatosis was evaluated using the SteatoTest (FibroMax panel), while fecal zonulin levels were measured by ELISA at baseline. Clinical, anthropometric, and metabolic parameters were assessed. We used ROC curve analysis to explore zonulin’s predictive value for moderate-to-severe steatosis (≥S2). *Results:* Elevated fecal zonulin (>107 ng/mL) occurred in 26.9% of participants. In a binary logistic model with SteatoTest ≥ S2 as outcome, zonulin was independently associated with clinically significant steatosis (OR per 1 ng/mL = 1.017; 95% CI 1.002–1.032; *p* = 0.029). Discrimination for ≥S2 was AUC = 0.680 (95% CI 0.535–0.825; *p* = 0.015). The Youden-optimal cut-off was 57.0 ng/mL (sensitivity 68.2%, specificity 63.3%) versus 40.9%/83.3% at the manufacturer’s 107 ng/mL threshold. *Conclusions*: Fecal zonulin shows modest discriminatory ability for steatosis and is best used as an adjunct to non-invasive assessment; cohort-specific calibration (57.0 ng/mL) outperformed the generic 107 ng/mL threshold.

## 1. Introduction

In recent decades, metabolic dysfunction-associated steatotic liver disease (MASLD) has emerged as a prominent focus of scientific investigation, driven by its escalating worldwide prevalence and its strong links to a broad spectrum of metabolic and systemic comorbidities. The condition carries a significant risk of progression to steatohepatitis, fibrosis, cirrhosis, or even hepatocellular carcinoma in the absence of timely medical or nutritional intervention [1]. The recent change in nomenclature reflects a deeper understanding of this disease, highlighting its links with insulin resistance, type 2 diabetes mellitus, dyslipidemia, cardiovascular disease, hypertension, and obesity [2]. The growing prevalence of obesity [3], as well as the increasing incidence of type 2 diabetes, cardiovascular disease, and dyslipidemia, has intensified scientific interest in the metabolic and clinical implications of MASLD [4]. Recent epidemiological assessments estimate that MASLD affects roughly one-third of adults worldwide (33–38%), with marked variation across geographic regions [5,6]. This prevalence is expected to rise further, paralleling the global increase in obesity, type 2 diabetes, and other metabolic disorders. In addition to its clinical impact, MASLD imposes a considerable socioeconomic burden, stemming from its chronic progression, the costs of advanced disease management, and associated losses in productivity [7].

Growing research has emphasized the pivotal role of the gut–liver axis in the pathogenesis of MASLD. This bidirectional communication pathway involves interactions between the intestinal microbiota, intestinal barrier integrity, and systemic inflammatory responses.

Among the proposed mechanisms, altered intestinal permeability—often referred to as “leaky gut”—has gained considerable attention. This condition allows the translocation of endotoxins, bacteria, and other pro-inflammatory molecules into the portal circulation, which may contribute to systemic inflammation, hepatic injury, and disease progression [8,9]. Lipopolysaccharides (LPS) derived from the intestinal microbiota can engage Toll-like receptor 4 (TLR4) on hepatic Kupffer cells, thereby initiating pro-inflammatory signaling cascades [10,11]. In parallel, gut microbial dysbiosis—marked by a depletion of short-chain fatty acid (SCFA)—producing taxa—may undermine mucosal immune function and compromise the integrity of the intestinal barrier [12].

Zonulin is an endogenous protein that modulates the function of intestinal tight junctions, thereby regulating the permeability of the gastrointestinal barrier. Elevated fecal concentrations have been documented in various metabolic conditions, including obesity and type 2 diabetes, as well as in multiple inflammatory and hepatic disorders, pointing to a potential association with impaired gut integrity [13,14]. Despite this, current evidence on its relevance in MASLD is scarce and often inconsistent, with only a limited number of studies specifically assessing its potential as a non-invasive marker of altered intestinal permeability in this patient population [14,15]. However, the contribution of this marker to MASLD pathophysiology remains poorly defined, as existing investigations have yielded inconsistent results [16]. Such discrepancies are likely attributable to heterogeneity in study cohorts, variability in diagnostic definitions, and differences in the analytical performance of commercially available enzyme-linked immunosorbent assay (ELISA) platforms [17,18].

This observational study aimed to evaluate fecal zonulin levels in patients with MASLD and to investigate their potential association with metabolic and hepatic parameters. We hypothesized that intestinal barrier dysfunction, reflected by elevated fecal zonulin, may be present in individuals with MASLD and could serve as a potential link between gut permeability and disease pathogenesis.

## 2. Materials and Methods

### 2.1. Study Design and Ethical Approval

The present investigation was conducted as a prospective, observational, single-center study and received ethical approval from the Ethics Committee of the Emergency Clinical County Hospital of Târgu Mureș (Approval No. Ad. 5004/16.02.2023). All participants provided written informed consent for inclusion in the study and granted permission for their data to be accessed exclusively for research purposes. All procedures were performed in full accordance with the ethical principles set forth in the Declaration of Helsinki.

The study was carried out from February to December 2023 and included 52 participants recruited from the Gastroenterology Department of the same institution. All patients were evaluated via abdominal ultrasonography, which confirmed the presence of hepatic steatosis.

Note on terminology: According to the 2023 international consensus, the terminology for fatty liver disease has been revised, with “non-alcoholic fatty liver disease (NAFLD)” now designated as “metabolic dysfunction-associated steatotic liver disease (MASLD)” and “non-alcoholic steatohepatitis (NASH)” redefined as “metabolic dysfunction-associated steatohepatitis (MASH).” In the present study, the terms NAFLD and NASH are applied where contextually appropriate, particularly when referring to established diagnostic methods, such as FibroMax and BARD, which were originally developed under the former classification framework.

The study was exploratory in nature and enrolled a consecutive sample of patients meeting the eligibility criteria; no formal sample size calculation was performed.

### 2.2. Inclusion and Exclusion Criteria

Participants eligible for inclusion were adults (≥18 years) with a confirmed diagnosis of hepatic steatosis or steatohepatitis, as established by abdominal ultrasonography and FibroMax testing. Enrollment was conducted consecutively in the Gastroenterology Department of the Emergency Clinical County Hospital of Târgu Mureș between February and December 2023.

Exclusion criteria comprised daily alcohol consumption above established guideline thresholds, lack of signed informed consent, and the presence of cirrhosis attributable to toxic or alcohol-related causes. Additional exclusion criteria were pregnancy or lactation, ongoing or planned bariatric surgery, psychiatric disorders, renal insufficiency, and advanced cardiovascular disease.

Hepatic steatosis on ultrasonography was defined as increased echogenicity of the liver parenchyma relative to the renal cortex, accompanied by ultrasound beam attenuation and reduced visualization of intrahepatic vessel walls.

### 2.3. Anthropometric and Biochemical Assessment

At baseline, all participants underwent a structured medical interview, including a detailed assessment of dietary habits and lifestyle. Anthropometric assessments, performed under standardized conditions, included measurements of height, weight, waist circumference, and hip circumference. Body mass index (BMI) was derived using the conventional formula (kg/m^2^). Measurements were performed by trained personnel blinded to the participants’ laboratory results. Fasting venous blood samples were collected following a minimum 12 h fast. The biochemical parameters measured included fasting plasma glucose (glycemia a jeun), the HOMA-IR index (Homeostasis Model Assessment of Insulin Resistance), liver function enzymes, total cholesterol, HDL cholesterol, serum triglycerides, and other routine laboratory indicators. To assess intestinal permeability, fecal samples were collected, and zonulin concentrations were determined using validated laboratory techniques in an accredited clinical laboratory. For the measurement of fecal zonulin, stool specimens were collected in sterile containers, immediately stored at 4 °C, and transported to the laboratory within 4 h of collection. Quantification was performed using a commercially available enzyme-linked immunosorbent assay (ELISA) kit (Immundiagnostik AG, Bensheim, Germany), following the specific procedural steps outlined in the manufacturer’s protocol. The minimum detectable concentration of the assay was 0.01 ng/mL, and the percentage variation observed within and between assay runs was below 8% and 10%, respectively. All measurements were performed in a laboratory certified under ISO 15189 quality standards. The ELISAs were performed by trained personnel at Bioclinica, independent from the study investigators. According to the manufacturer’s instructions, a fecal zonulin concentration ≤ 107 ng/mL was considered within the normal range, while values > 107 ng/mL were classified as elevated. These categories were used for patient stratification in subsequent statistical analyses (Table 1).

### 2.4. Hepatic Evaluation: FibroMax and BARD Score

All patients underwent hepatic evaluation using the FibroMax test panel, a non-invasive diagnostic tool developed by BioPredictive (Paris, France) and validated in multiple clinical studies for the assessment of NAFLD and NASH. The FibroMax panel includes five distinct components, each designed to evaluate a specific aspect of liver pathology: FibroTest assesses the degree of fibrosis, ActiTest measures necro-inflammatory activity, SteatoTest estimates the extent of steatosis, NashTest screens for non-alcoholic steatohepatitis, and AshTest helps differentiate alcohol-related hepatic injury.

Interpretation of the results was based on reference standards provided by the manufacturer. For FibroTest, scores ranged from F0 (no fibrosis) to F4 (severe fibrosis). SteatoTest scores were classified from S0 (no steatosis) to S3 (advanced steatosis). ActiTest scores ranged from A0 (no necro-inflammatory activity) to A3 (severe activity). NashTest scores ranged from N0 (no inflammation) to N2 (confirmed inflammation), and AshTest scores ranged from H0 (no alcoholic inflammation) to H3 (severe alcoholic inflammation).

The BARD score was calculated for each participant to estimate the likelihood of advanced fibrosis and cirrhosis in individuals with non-alcoholic fatty liver disease (NAFLD). This scoring system, ranging from 0 to 4, incorporates three clinical parameters: body mass index (BMI), abdominal obesity, and the presence of type 2 diabetes. It is commonly used to estimate the risk of advanced fibrosis in metabolic liver disease. Based on BMI, participants were divided into three groups: Group 0 (normal BMI < 25 kg/m^2^), Group 1 (overweight, BMI 25.0–29.9 kg/m^2^), and Group 2 (obese, BMI ≥ 30 kg/m^2^).

The BARD score was computed as per the criteria established in the initial description by Harrison et al., where a BMI ≥ 28 kg/m^2^ scores 1 point, a diagnosis of type 2 diabetes mellitus scores 1 point, and an AST/ALT ratio ≥ 0.8 scores 2 points. The total score ranges from 0 to 4, with higher values indicating an increased probability of advanced fibrosis.

### 2.5. Statistical Analysis

All statistical analyses were carried out using IBM SPSS Statistics software, version 21.0 (IBM Corp., Armonk, NY, USA), and graphical outputs were created in Microsoft Excel. Group comparisons included stratification according to fecal zonulin categories, as defined above (≤107 ng/mL vs. >107 ng/mL). Data distribution was examined with the Shapiro–Wilk test. For age and BMI, data followed a normal distribution (*p* > 0.05), whereas HOMA-IR values deviated from normality (*p* < 0.05). Continuous variables are reported as mean ± standard deviation (SD), and categorical variables as absolute counts and percentages.

Between-group differences were analyzed using the independent-samples Student’s *t*-test for normally distributed continuous data and the chi-square (χ^2^) test for categorical data. Correlations between variables were assessed using Spearman’s rank correlation coefficient (ρ) for ordinal or non-normally distributed variables (e.g., SteatoTest stage, BARD score), while Pearson’s correlation was reserved for approximately normally distributed continuous pairs.

For continuous variables that did not follow a normal distribution, the Mann–Whitney U test was applied. Multivariate logistic regression analyses were conducted to control for variables that might confound the associations, including age, sex, BMI, and diagnosis of type 2 diabetes mellitus, with results reported in terms of odds ratios (ORs) and 95% confidence intervals (CIs).

The diagnostic accuracy of fecal zonulin for predicting moderate-to-severe steatosis (SteatoTest ≥ S2) was evaluated using receiver operating characteristic (ROC) curve analysis, with calculation of the area under the curve (AUC). The ROC analysis was conducted on the full study cohort (*n* = 52), for whom complete paired data on fecal zonulin concentrations and SteatoTest results were available. The outcome distribution comprised 31 patients with SteatoTest ≥ S2 and 21 patients with SteatoTest < S2, meeting published methodological recommendations for stable AUC estimation in diagnostic accuracy studies. The 95% confidence interval for the AUC was computed using the Hanley–McNeil method, and the null hypothesis AUC = 0.50 was tested with a two-sided z test (α = 0.05). A two-sided *p*-value of <0.05 was considered to indicate statistical significance. Beyond the manufacturer’s threshold (≤107 ng/mL), we first applied SPSS CATREG (optimal-scaling categorical regression) with SteatoTest category (<S2 vs. ≥S2) as the dependent variable and zonulin (ng/mL) entered as an ordinal predictor to explore a cohort-specific cut-off. For interpretable effect sizes, we then fitted a companion binary logistic regression and report odds ratios (ORs) with 95% CIs; the optimal cut-off was chosen by maximizing Youden’s index. No missing data were recorded for the variables included in the statistical analysis.

## 3. Results

### 3.1. Baseline Characteristics Stratified by Fecal Zonulin Levels

Of the 52 patients diagnosed with MASLD who were included in the final analysis, 30 (57.7%) were women, with a mean age of 43.7 ± 13.2 years and a mean body mass index of 28.6 ± 5.2 kg/m^2^. Elevated fecal zonulin levels (>107 ng/mL), indicative of increased intestinal permeability, were observed in 14 patients (26.9%).

When comparing individuals with elevated vs. normal zonulin levels, certain metabolic differences emerged, though statistical significance was not reached. Patients with higher zonulin values had, on average, a higher BMI (30.4 ± 5.6 vs. 27.9 ± 4.8 kg/m^2^; *p* = 0.12) and a greater proportion of insulin resistance as defined by HOMA-IR ≥ 2.5 (57.1% vs. 34.2%; *p* = 0.18). (Table 1). Notably, moderate-to-severe hepatic steatosis (SteatoTest ≥ S2) was more frequent among patients with elevated zonulin (78.6%) compared to those with lower levels (55.3%), reflecting a potential trend (*p* = 0.10). No significant differences were observed regarding age, sex distribution, or fibrosis stage (FibroTest ≥ F1) between the two groups. Specifically, the proportion of female patients was 57.9% vs. 57.1%, and male patients 42.1% vs. 42.9% in the normal and elevated zonulin groups, respectively.

### 3.2. Non-Invasive Hepatic Profiling Using the FibroMax Panel

A comprehensive non-invasive hepatic evaluation was performed for all participants using the FibroMax panel, which includes SteatoTest, FibroTest, ActiTest, and NashTest (Table 2).

Hepatic steatosis was highly prevalent, with 80.8% of patients presenting with at least mild steatosis (≥S1), and 34.5% exhibiting either moderate (S2) or severe (S3) steatosis. An additional 17.3% displayed intermediate stages (S1–S2 or S2–S3), which may reflect transitional metabolic profiles.

Regarding liver fibrosis, 36.5% of participants had any degree of fibrosis (≥F1), while 5.8% had advanced fibrosis (F3–F4). Necro-inflammatory activity (ActiTest ≥ A1) was observed in 40.4% of patients, including 17.3% with moderate-to-severe activity. Additionally, 11.5% of patients were classified with definite NASH (N2), while 40.4% were borderline (N1), suggesting potential inflammatory liver injury in a substantial proportion of the cohort.

These findings support the metabolic burden and hepatic involvement associated with MASLD, even in a relatively young and ambulatory population.

### 3.3. Correlation Between Fecal Zonulin and Hepatic or Metabolic Parameters

**Figure 1:**
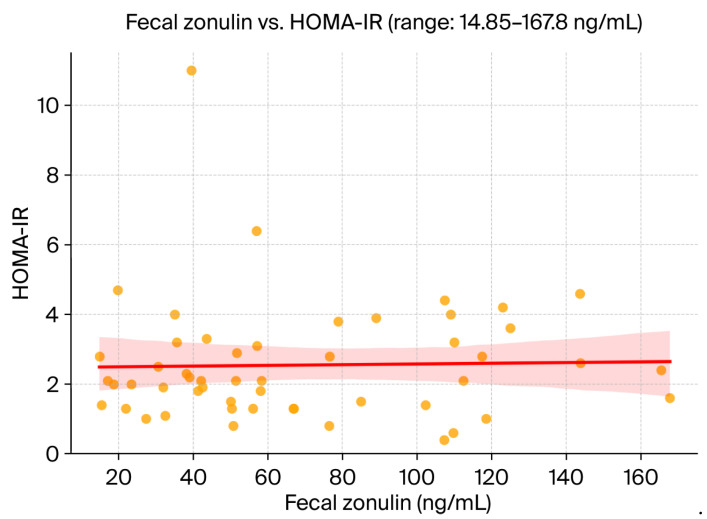
Scatterplot illustrating the relationship between fecal zonulin concentrations and insulin resistance (HOMA-IR). The solid red line indicates the fitted regression with its 95% confidence interval. No statistically significant relationship was observed (ρ = 0.092, *p* > 0.05).

Spearman’s correlation analysis revealed a modest, non-significant positive association between fecal zonulin concentrations and hepatic steatosis severity, as assessed by the SteatoTest (ρ = 0.162). No meaningful correlations were observed between fecal zonulin and FibroTest-derived fibrosis scores (ρ = 0.011) or insulin resistance as measured by HOMA-IR (ρ = 0.092). These findings suggest that, within this cohort, zonulin levels did not strongly reflect hepatic fibrotic burden or systemic metabolic dysfunction. A scatterplot of fecal zonulin versus HOMA-IR is provided in Figure 1.

### 3.4. Predictive Value of Fecal Zonulin for Hepatic Steatosis

**Figure 2:**
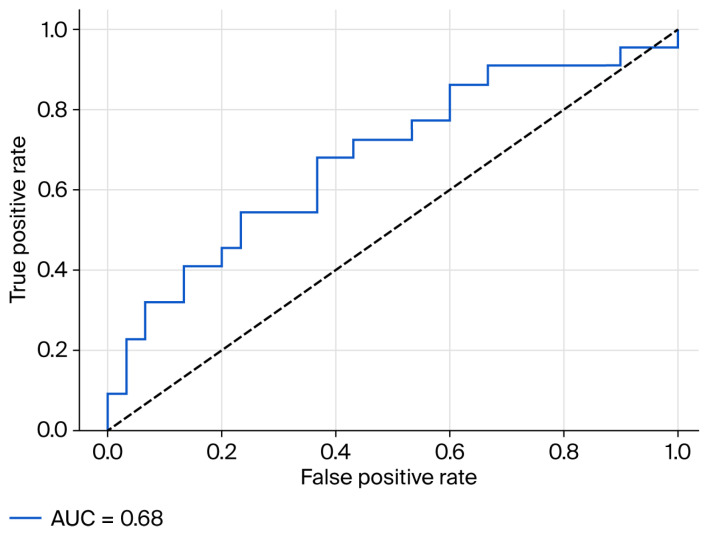
ROC Curve for Fecal Zonulin Predicting Moderate-to-Severe Steatosis (SteatoTest ≥ S2). AUC = 0.680 (95% CI: 0.535–0.825), *p* = 0.015. Youden-optimal cut-off = 57.0 ng/mL (sensitivity 68.2%, specificity 63.3%).

To better evaluate the potential clinical utility of fecal zonulin in MASLD, we examined its ability to distinguish cases of moderate-to-severe hepatic steatosis (SteatoTest ≥ S2) through receiver operating characteristic (ROC) analysis. The resulting area under the curve (AUC) was 0.68 (95% CI: 0.535–0.825; *p* = 0.015), suggesting a moderate–though possibly relevant–capacity of fecal zonulin to differentiate between patients with and without clinically significant steatosis (Figure 2). This analysis included the entire study population (*n* = 52), all of whom had complete fecal zonulin and SteatoTest data. Among them, 31 patients met the criterion for moderate-to-severe steatosis (SteatoTest ≥ S2), while 21 did not (SteatoTest < S2). These group sizes are consistent with methodological standards for ROC curve construction, ensuring adequate stability of the AUC estimate.

Although the diagnostic performance did not reach thresholds typically associated with standalone biomarkers, the observed AUC suggests a potential contributory role for fecal zonulin when interpreted alongside other clinical and biochemical parameters. Given its non-invasive nature and biological plausibility as a marker of intestinal permeability, fecal zonulin may warrant further evaluation in larger, prospective cohorts and in multimarker predictive models.

### 3.5. Optimized Cut-Off for Fecal Zonulin

Using SPSS CATREG with optimal scaling, fecal zonulin showed a positive monotonic association with SteatoTest ≥ S2. In the companion binary logistic model, the per-ng/mL effect corresponded to OR = 1.017 (95% CI 1.002–1.032; *p* = 0.029). Maximizing Youden’s index identified an optimal threshold of 57.0 ng/mL (sensitivity 68.2%, specificity 63.3%); at 107 ng/mL, sensitivity was 40.9% and specificity 83.3%. Discrimination was quantified by the ROC curve (AUC as reported in 3.4 subsection). Maximizing Youden’s index identified an optimal threshold of 57.0 ng/mL (sensitivity 68.2%, specificity 63.3%). At the manufacturer’s threshold of 107 ng/mL, sensitivity was 40.9% and specificity was 83.3%. These data indicate that a cohort-calibrated cut-off improves overall classification relative to the generic reference value.

### 3.6. BARD Score Analysis

To evaluate the clinical validity of the BARD score within this MASLD cohort, we examined monotonic associations using Spearman’s rank correlation (ρ, two-tailed). BARD correlated with body mass index (BMI) (ρ = 0.342, *p* < 0.05) and with hepatic steatosis severity assessed by the SteatoTest (ρ = 0.334, *p* < 0.05). As expected, the correlation between BMI and SteatoTest was stronger (ρ = 0.638, *p* < 0.001), consistent with the impact of adiposity on hepatic fat accumulation. These findings support the utility of BARD for metabolic risk stratification in MASLD, with small-to-moderate effect sizes in this relatively young, ambulatory cohort. No relevant correlations were observed between BARD score and fecal zonulin, suggesting that gut permeability–as captured by zonulin–represents a complementary biological dimension not embedded in BARD (Table 3).

## 4. Discussion

### 4.1. Intestinal Barrier Dysfunction and Its Role in MASLD Pathogenesis

Compromise of the intestinal barrier is increasingly recognized as a pivotal factor in the development of metabolic dysfunction-associated steatotic liver disease (MASLD). Zonulin, a physiological modulator of tight junctions, reflects gut permeability and has been increasingly studied as a non-invasive biomarker. Recent investigations have shown elevated zonulin levels in MASLD and non-alcoholic fatty liver disease (NAFLD), particularly in relation to steatosis rather than fibrosis [14,19]. In our prospective cohort, 26.9% of patients demonstrated elevated fecal zonulin (>107 ng/mL). While the correlation between zonulin levels and SteatoTest score was modest (ρ = 0.162, *p* = 0.287), the noticeably higher prevalence of moderate-to-severe steatosis in this subgroup (78.6% vs. 55.3%) raises the possibility that microbial translocation through a disrupted gut barrier may contribute to systemic inflammatory responses and promote fat deposition within the liver [19,20,21]. In a categorical regression, fecal zonulin was independently associated with SteatoTest ≥ S2 (OR per 1 ng/mL = 1.017; 95% CI 1.002–1.032; *p* = 0.029). Although the bivariate correlation did not reach statistical significance, this effect direction was concordant with increased intestinal permeability in MASLD and supports external validation in larger, controlled cohorts.

### 4.2. Zonulin, Fibrosis, and Insulin Resistance

Fecal zonulin was not significantly associated with liver fibrosis severity (FibroTest, ρ = 0.011, *p* = 0.944) or insulin resistance (HOMA-IR, ρ = 0.092, *p* = 0.549). This finding supports the hypothesis that increased intestinal permeability may operate early and independently in MASLD pathogenesis, preceding both fibrotic remodeling and systemic metabolic dysfunction [14,22,23]. These results mirror recent data indicating that gut barrier dysfunction tends to be more pronounced in early-stage steatosis and may evolve independently from traditional metabolic markers such as insulin resistance.

### 4.3. Diagnostic Utility of Zonulin

The discriminative ability of fecal zonulin for identifying moderate-to-severe steatosis was modest (AUC = 0.68, CI 0.535–0.825; *p* = 0.015), in line with earlier reports that caution against using zonulin as a standalone diagnostic tool [24]. However, given its non-invasive nature and plausible biological role, zonulin may hold added value in multimodal assessment frameworks. Integrative diagnostic strategies that combine permeability markers (zonulin, LBP), microbial-derived metabolites, and established metabolic indices have shown promise in improving MASLD stratification [14,25]. In this context, fecal zonulin could contribute to refining risk profiles, particularly in patients with ambiguous clinical or imaging findings.

### 4.4. The BARD Score and Gut–Liver Axis Disconnection

The BARD score remains a practical fibrosis risk assessment tool, based on simple clinical parameters: BMI, presence of type 2 diabetes, and AST/ALT ratio. In our study, BARD score correlated with BMI (ρ = 0.342, *p* < 0.05) and SteatoTest (ρ = 0.334, *p* < 0.05), confirming its dependence on metabolic components. However, no significant correlation was observed between BARD score and fecal zonulin. This dissociation highlights that gut barrier dysfunction may reflect a distinct axis of MASLD pathophysiology—one not captured by conventional metabolic risk scores [15,26]. Therefore, there is growing rationale to develop enhanced predictive models that incorporate gut-derived biomarkers alongside traditional tools such as the BARD, FIB-4, or FAST scores.

### 4.5. Pathophysiological Basis and Experimental Evidence

Evidence from preclinical research supports the biological plausibility of an association between altered intestinal barrier function and liver injury. Experimental studies in animal models have shown that decreased expression of tight junction proteins like ZO-1 and occludin can lead to increased permeability, allowing microbial compounds such as LPS to reach the liver and trigger inflammatory processes and lipid buildup [27,28]. Dysbiosis-induced activation of the toll-like receptor 4 (TLR4) pathway has also been shown to exacerbate hepatic steatosis and fibrosis [29,30]. Our findings are consistent with this gut–liver axis framework and provide clinical evidence supporting its translational relevance in human MASLD.

### 4.6. Clinical Relevance

Despite the moderate strength of associations, fecal zonulin remains a biologically plausible and easily obtainable biomarker that may help capture a previously underrecognized component of MASLD: intestinal permeability. Its incorporation into non-invasive diagnostic algorithms—especially in combination with other stool-based or serum markers—could enrich risk stratification, particularly for patients in early or ambiguous stages of the disease.

Future research should pursue multicenter validation in larger cohorts, standardized fecal biomarker collection protocols, and head-to-head comparisons with alternative gut-derived markers (e.g., calprotectin, LBP). Longitudinal studies are also needed to evaluate whether changes in zonulin over time correlate with progression or regression of hepatic steatosis and fibrosis, particularly in response to lifestyle or pharmacologic interventions.

### 4.7. Study Strengths and Limitations

This study’s prospective design, use of the validated FibroMax panel, and inclusion of zonulin as a non-invasive intestinal permeability biomarker are noteworthy strengths. Moreover, the use of SteatoTest enables nuanced stratification of steatosis stages, correlating with histologic findings in previous validation studies. Nonetheless, some limitations must be acknowledged. The relatively small sample size (*n* = 52), single-center recruitment, and absence of a healthy control group restrict generalizability. In addition, variability in stool consistency and pre-analytical handling can affect fecal zonulin reliability [31]. These challenges highlight the need for technical standardization and external validation in future studies.

Taken together, our results indicate that alterations in intestinal permeability may play a contributory role during the initial phases of MASLD, highlighting the value of integrating gut–liver axis biomarkers into non-invasive evaluation approaches. While zonulin alone may not offer sufficient diagnostic accuracy, its inclusion in multimodal frameworks holds promise for enhancing early detection, risk stratification, and personalized care in MASLD.

### 4.8. Perspectives and Future Directives

The present findings open several avenues for further exploration of the gut–liver axis in MASLD. First, validation in larger, ethnically diverse, multicenter cohorts is essential to confirm the reproducibility of our results and to evaluate potential geographic or lifestyle-related variability in zonulin levels. Second, the integration of fecal zonulin with complementary biomarkers—such as circulating lipopolysaccharide-binding protein (LBP), microbial metabolites, or advanced imaging parameters—could yield multimodal diagnostic algorithms with higher discriminatory capacity than single-marker approaches

Longitudinal studies are particularly warranted to determine whether dynamic changes in zonulin concentrations reflect hepatic disease progression or regression, especially in response to targeted dietary, microbiome-directed, or pharmacological interventions. In parallel, mechanistic studies linking intestinal permeability alterations to specific microbial taxa, metabolomic profiles, and inflammatory pathways could provide deeper insight into causal relationships and identify therapeutic targets.

Finally, the development of standardized pre-analytical and analytical protocols for fecal biomarker assessment—including sample collection, storage, and assay harmonization—will be critical to ensure comparability across studies and to enable clinical translation. By addressing these directions, future research may clarify the role of gut barrier dysfunction in MASLD pathophysiology and determine whether zonulin measurement can transition from a research tool to a clinically actionable parameter.

## 5. Conclusions

Fecal zonulin indexes an intestinal-permeability domain in MASLD that aligns with clinically relevant steatosis but not with fibrosis, insulin resistance, or BARD. Cohort-specific calibration using categorical regression with ROC optimization improved classification over the manufacturer threshold, yet stand-alone performance remains modest. Accordingly, zonulin is best deployed as an adjunct within standardized, externally validated multimarker algorithms.

## Figures and Tables

**Table 1:** Baseline clinical and metabolic characteristics according to fecal zonulin levels in MASLD patients (*n* = 52).

Variable	All Patients (*n* = 52)	Zonulin ≤ 107 ng/mL (*n* = 38)	Zonulin > 107 ng/mL (*n* = 14)	*p*-Value
Age (years)	43.7 ± 13.2	42.1 ± 12.5	47.8 ± 14.2	0.15
Female, *n* (%)	30 (57.7%)	22 (57.9%)	8 (57.1%)	0.95
Male, *n* (%)	22 (42.3%)	16 (42.1%)	6 (42.9%)	0.95
BMI (kg/m^2^)	28.6 ± 5.2	27.9 ± 4.8	30.4 ± 5.6	0.12
HOMA-IR ≥ 2.5, *n* (%)	21 (40.4%)	13 (34.2%)	8 (57.1%)	0.18
SteatoTest ≥ S2, *n* (%)	31 (59.6%)	21 (55.3%)	11 (78.6%)	0.10
FibroTest ≥ F1, *n* (%)	16 (30.8%)	10 (26.3%)	5 (31.3%)	0.73

Note: Values are expressed as mean ± SD or *n* (%). Comparisons were made using Student’s *t*-test for continuous variables and χ^2^ test for categorical variables. HOMA-IR ≥ 2.5 defines insulin resistance; SteatoTest ≥ S2 indicates moderate-to-severe steatosis; FibroTest ≥ F1 indicates presence of liver fibrosis. Age and BMI met normality assumptions (Shapiro–Wilk *p* > 0.05); HOMA-IR did not (*p* < 0.05).

**Table 2:** Distribution of SteatoTest, FibroTest, ActiTest, and NashTest Scores at Baseline in Patients with MASLD (*n* = 52).

Score Type	Category	*n* (%)
SteatoTest	S0 (No steatosis)	10 (19.2%)
	S1 (Mild)	15 (28.8%)
	S2 (Moderate)	8 (15.4%)
	S3 (Severe)	10 (19.2%)
	Intermediate (S1–S2, S2–S3)	9 (17.3%)
FibroTest	F0 (No fibrosis)	33 (63.5%)
	F1 (Mild)	6 (11.5%)
	F2 (Moderate)	5 (9.6%)
	F3–F4 (Advanced)	3 (5.8%)
	F4	5 (9.6%)
ActiTest	A0 (No necro-inflammatory activity)	31 (59.6%)
	A1 (Mild activity)	12 (23.1%)
	A2–A3 (Moderate–Severe activity)	9 (17.3%)
NashTest	N0 (No inflammation)	25 (48.1%)
	N1 (Possible NASH)	21 (40.4%)
	N2 (Definite NASH)	6 (11.5%)

Note: Intermediate SteatoTest stages (e.g., S1–S2, S2–S3) were grouped to reflect clinical ambiguity in biomarker transitions. All percentages are calculated from the total study population (*n* = 52). Minor deviations from 100% are due to rounding.

**Table 3:** Spearman Rank Correlation Matrix Between BMI, SteatoTest, and BARD Score (*n* = 52).

	BMI	SteatoTest	BARD Score
BMI	1	0.638 **	0.342 *
SteatoTest	0.638 **	1	0.334 *
BARD score	0.342 *	0.334 *	1

Values represent Spearman rank correlation coefficients (ρ). All correlations marked ** are significant at the 0.01 level (two-tailed), and all correlations marked * are significant at the 0.05 level (two-tailed).

## Data Availability

Data are contained within the article.

## Abbreviations

The following abbreviations are used in this manuscript:

MASLD	Metabolic dysfunction-associated steatotic liver disease
NAFLD	Non-alcoholic fatty liver disease
NASH	Non-alcoholic steatohepatitis
MASH	Metabolic dysfunction-associated steatohepatitis
BMI	Body mass index
T2DM	Type 2 diabetes mellitus
HOMA-IR	Homeostasis Model Assessment of Insulin Resistance
AUC	Area under the curve
ROC	Receiver operating characteristic
LPS	Lipopolysaccharide
ZO-1	Zonula occludens-1
AST	Aspartate aminotransferase
ALT	Alanine aminotransferase
HDL	High-density lipoprotein
LDL	Low-density lipoprotein
LBP	Lipopolysaccharide-binding protein
ELISA	Enzyme-linked immunosorbent assay
SPSS	Statistical Package for the Social Sciences
SD	Standard Deviation
